# Combined immunotherapy with anti-PDL-1/PD-1 and anti-CD4 antibodies cures syngeneic disseminated neuroblastoma

**DOI:** 10.1038/s41598-017-14417-6

**Published:** 2017-10-25

**Authors:** Valentina Rigo, Laura Emionite, Antonio Daga, Simonetta Astigiano, Maria Valeria Corrias, Concetta Quintarelli, Franco Locatelli, Silvano Ferrini, Michela Croce

**Affiliations:** 1 Dipartimento di terapie oncologiche integrate, IRCCS A.O.U. San Martino-IST, Istituto Nazionale per la Ricerca sul Cancro, Largo R. Benzi 10, 16132 Genova, Italy; 2 Dipartimento della diagnostica, della patologia e delle cure ad alta complessità tecnologica, IRCCS A. O. U. San Martino-IST, Istituto Nazionale per la Ricerca sul Cancro, Largo R. Benzi 10, 16132 Genova, Italy; 3 Dipartimento Ricerca Traslazionale, Medicina di Laboratorio, Diagnostica e Servizi, IRCCS Istituto Giannina Gaslini, L.go G. Gaslini 5, 16147 Genova, Italy; 4 Dipartimento di Oncoematologia Pediatrica, IRCCS Ospedale Pediatrico Bambino Gesù, Roma, Italy; 5 Dipartimento di Medicina Clinica e Chirurgia, Università di Napoli Federico II, Napoli, Italy; 6 Dipartimento di Scienze Pediatriche, Università di Pavia, Pavia, Italy

## Abstract

Anti-PD-1 or anti-PD-L1 blocking monoclonal antibodies (mAbs) have shown potent anti-tumor effects in adult cancer patients and clinical studies have recently been started in pediatric cancers, including high-risk/relapsing neuroblastoma (NB). Therefore, we studied the effects of anti-PD-1/PD-L1 mAbs in two syngeneic models of disseminated NB generated by the injection of either Neuro2a or NXS2 cells, which express PD-L1. In addition, we tested the combination of these agents with the immune-enhancing cytokine IL-21, the Ecto-NTPDase inhibitor POM-1, an anti-CD25 mAb targeting Treg cells, or an anti-CD4 mAb. We previously showed that CD4-transient depletion removes CD4^+^CD25^+^ Treg cells and other CD4^+^CD25^−^ regulatory subsets. Here we show that mono-therapy with anti-PD-1/PD-L1 mAbs had no effect on systemic NB progression *in vivo*, and also their combination with IL-21, POM-1 or anti-CD25 mAb was ineffective. The combined use of anti-PD-1 with an anti-CD4 mAb mediated a very potent, CD8-dependent, synergistic effect leading to significant elongation of tumor-free survival of mice, complete tumor regression and durable anti-NB immunity. Similar results were obtained by combining the anti-PD-L1 and anti-CD4 mAbs. These findings indicate that both PD-1/PD-L1 and CD4^+^ T cell-related immune-regulatory mechanisms must be simultaneously blocked to mediate therapeutic effects in these models.

## Introduction

Immune checkpoints are fundamental for the physiological maintenance of tolerance and protection of tissues from the damage that an unregulated immune response may cause. It is now clear that immune checkpoint dysregulation represents an important mechanism through which tumors escape immune system recognition and progress^1^. Studies in preclinical tumor models and cancer patients have proven that the targeting of immune checkpoints restores a silenced anti-tumor immune response, which then becomes effective in eliminating tumor cells. Indeed, the monoclonal antibodies (mAbs) blocking immune checkpoints such as Cytotoxic T-Lymphocyte Antigen (CTLA)-4 and Programed Death-Ligand1 (PD-L1)/Programed Death-1 (PD-1) showed extraordinary anti-tumor effects in clinical trials leading to their approval as anti-cancer drugs in different metastatic cancers^2^. In particular, anti-PD-1 mAbs have shown unprecedented clinical activity in patients with metastatic melanoma^3–8^, non-small-cell lung carcinoma (NSCLC)^9,10^ and renal cell carcinoma^11^, with a significant fraction of patients showing prolonged clinical benefit. The combination of anti-PD-1 and anti-CTLA-4 leads to a further increase in clinical responses in metastatic melanoma^12^. Synergistic therapeutic activity may relate to the different site of action of CTLA4 and PD-L1/PD-1-blocking agents, i.e. the secondary lymphoid organs and the peripheral tissues, respectively. In addition, CTLA-4 blockade induces a proliferative gene expression signature predominantly in a subset of transitional memory T cells, whereas PD-1 blockade leads to changes in genes implicated in cytolysis^13^. However, the combination of anti-CTLA-4 and anti-PD-1 mAbs also results in increased toxicities in a significant fraction of treated patients^12^. Therefore, the development of less toxic anti-PD-1-based combination therapies is an important area of research, and different combinations are currently being investigated in clinical trials^14,15^. In view of their activity in different cancers, mAbs targeting PD-L1 (NCT02541604), or PD-1 (NCT02332668, NCT02304458) alone or combined with other mAbs (NCT02304458; NCT02914405), are also being tested in pediatric solid cancers, including relapsing Neuroblastoma (NB). NB is the most common extra-cranial solid tumor of childhood and is a heterogeneous disease, comprising forms that undergo spontaneous regression and forms that can rapidly progress to a fatal outcome, such as high-risk, stage-4 NB^16^. About 50% of cases at diagnosis is represented by stage-4 NB, which in spite of aggressive treatment, has survival rates of approximately 40% at 5 years^17^. Immunotherapy may represent an additional tool that may be combined with existing therapies in refractory or relapsing NB^18^.

We previously showed that immunotherapy with the immune-enhancing cytokine IL-21^19^ in combination with an anti-CD4 mAb was highly effective in a syngeneic model of disseminated NB, through the activation of a cytotoxic T-lymphocyte (CTL) response^20,21^. In this NB model, tumor progression leads to an increase of CD4^+^CD25^+^FoxP3^+^ Treg cells, although also CD4^+^CD25^−^ T cells mediated immune regulatory functions, as the use of anti-CD25 mAb was ineffective^20^.

Here, we show that, although the murine NB cell lines Neuro2a and NXS2 express PD-L1, the use of anti-PD-1 or PD-L1 mAb has no impact on progression of disseminated NB, in syngeneic mice. We therefore explored the possible cooperative effects of anti-PD-1 mAb combined with either IL-21, as immune enhancing agent, or with an anti-CD25 mAb or with the NTPDase-inhibitor POM-1, which target Treg cells, or with a cell-depleting anti-CD4 mAb, which transiently depletes the whole CD4^+^ T-cell population.

## Results

### Anti-PD-1/PD-L1 mAb alone or their combinations with rIL-21 have no impact on the progression of PD-L1-expressing NB in syngeneic mice

**Figure 1:**
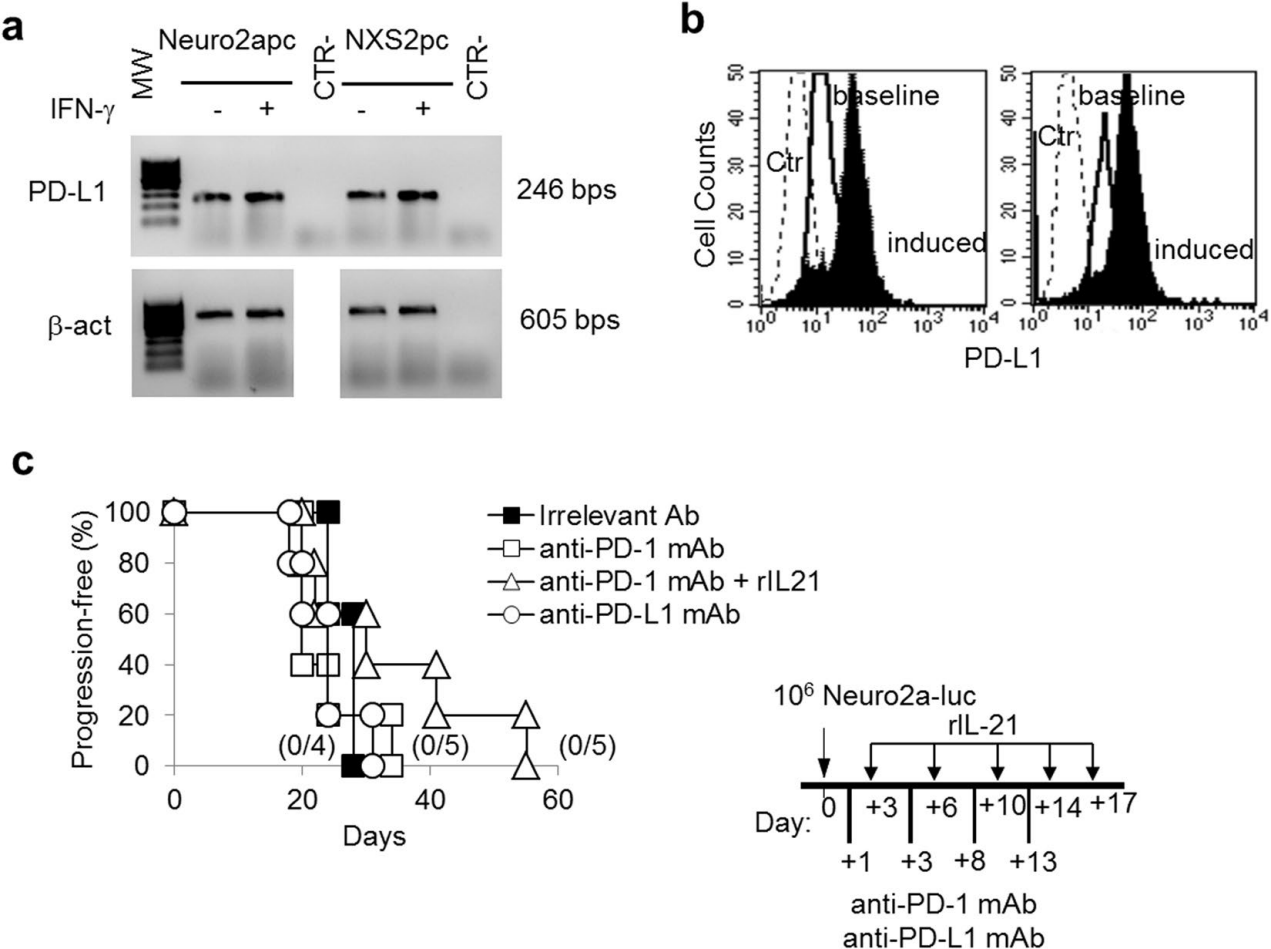
Neuro2a cells express PD-L1 but anti-PD-1 or anti-PD-L1 blocking mAbs have no impact on Neuro2a tumor progression in syngeneic mice. (**a**) RT-PCR analysis shows that Neuro2apc and NXS2pc cells express PD-L1 mRNA constitutively and after 48 hrs IFN-γ treatment (1,000 IU/ml). Housekeeping gene was β-actin. β-actin gel was cropped from the same gel to uniform the loading order to PD-L1 gel. MW: molecular weight marker 100 bp ladder DNA. CTR-: PCR negative control. (**b**) Neuro2a and NXS2 cells express surface PD-L1 as detected by immunofluorescence and FACS analysis. Ctr: isotype control PE; baseline: cells stained with anti-PD-L1 PE; induced: cells treated with 1,000 IU/ml rat IFN-γ for 48 hrs and stained with anti-PD-L1 PE. (**c**) Kaplan-Meier analysis of A/J mice inoculated i.v. with a tumorigenic dose of Neuro2a-luc cells on day 0 and treated with an irrelevant antibody, anti-PD-1, anti-PD-L1 mAb or with anti-PD-1 combined with rIL-21, according to the schedule shown in the inset. Percentages of progression-free mice are indicated on the Y-axis and the fraction of progression-free mice of each group is given in brackets.

Tumor cells may express PD-L1 either constitutively or because of exposure to cytokines, such as IFN-γ or IL-27^1,22,23^. In the case of murine Neuro2a and NXS2 NB cell lines, RT-PCR and immunofluorescence analyses indicate that these cells express PD-L1 constitutively (Fig. 1a and b, respectively). Similarly to some human NB cells^22^, surface PD-L1 expression is further induced by IFN-γ treatment, in murine NB cells. We first used the Neuro2a-luc model of disseminated syngeneic NB for testing PD-1/PD-L1 checkpoint blockers, either alone or in combination with other immune-enhancing agents. An anti-PD-1 blocking mAb was administered i.p. in a 4-dose schedule, starting from the day after NB injection (Fig. 1c). Different from other syngeneic tumor models^24^, the anti-PD-1 mAb alone had no effect and all treated mice developed disseminated tumors similar to irrelevant mAb-treated control mice (Fig. 1c), as detected by IVIS (Supplementary Figure S1). In addition, also immunotherapy using an anti-PD-L1 blocking mAb had no impact on Neuro2a tumor growth (Fig. 1c). Similar results were obtained in the GD2^+^ NXS2 model (Supplementary Figure S2).

In the search of possible cooperative/synergistic effects with other immune-enhancing agents, we combined anti-PD-1 mAb with rIL-21. Our previous data indicated that repeated s.c. injections of rIL-21 have a limited anti-tumor activity in the Neuro2a model, although the combination with an anti-CD4 mAb showed a potent synergistic effect^21^. However, the combined treatment with anti-PD-1 blocking mAb and rIL-21 showed a modest (p = n.s.) increase in progression-free survival, relative to anti-PD-1 alone, since all mice finally developed progressive disease (Fig. 1c and Supplementary Figure S1). Also, the anti-PD-L1 blocking mAb had no cooperative effect with rIL-21. Similarly, rIL-21 failed to cooperate with anti-PD-L1 or anti-PD-1 mAbs in the NXS2 model (Supplementary Figure S2).

### Combination therapy with anti-PD-1/PD-L1 plus anti-CD4 mAb showed synergistic activity and cured mice from disseminated NB

**Figure 2:**
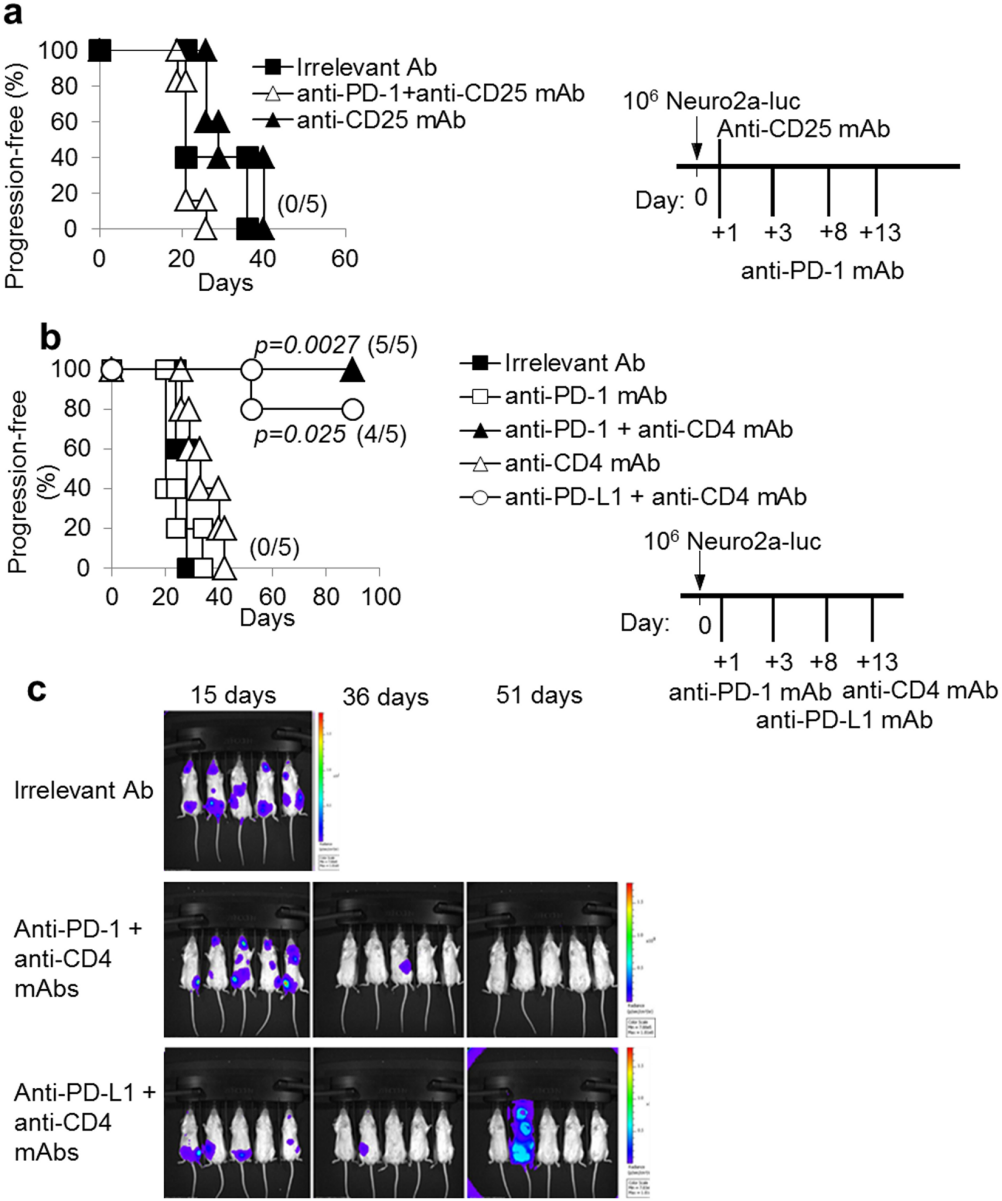
The combination of anti-PD-1 or anti-PD-L1 with a cell-depleting anti-CD4 mAb inhibits NB progression. (**a**) Kaplan-Meier analysis of A/J mice challenged i.v. with a tumorigenic dose of Neuro2a-luc cells on day 0 and then treated with anti-CD25 mAb alone or combined with anti-PD-1 mAb or with an irrelevant antibody, according to the schedule shown in the inset. Percentages of progression-free mice are indicated on the Y-axis and the fraction of progression-free mice of each group is given in brackets. (**b**) The combined treatment with anti-PD-1 or anti-PD-L1 and anti-CD4 mAb potently inhibits NB progression. P values of combined treatments vs irrelevant Ab controls are indicated (Wilcoxon log-rank test). The experiment shown is representative of two independent ones with identical results. Percentages of progression-free mice are indicated on the Y-axis and the fraction of progression-free mice of each group is given in brackets. (**c**) IVIS analysis representation of Neuro2a-luc NB development in mice receiving irrelevant Ab and combined treatments at the indicated time-points from challenge.

Our previous study indicated that i.v. injection of a tumorigenic dose of Neuro2a cells induces a significant increase of CD4^+^CD25^+^Foxp3^+^ Treg cells, in the spleen and lymph nodes. These Treg cells may impair the anti-tumor immune response and limit the efficacy of immunotherapy^20^. One of the mechanisms used by Treg cells to suppress immune responses is the production of adenosine^25^ through the activity of the Ecto-NTPDases CD39 and CD73. Therefore, we treated Neuro2a-luc-bearing mice with the NTPDase inhibitor POM-1 alone or in combination with anti-PD-1 mAb. However, these treatments were ineffective on progression-free survival of mice (Supplementary Figure S3). Also, a cell-depleting anti-CD25 mAb, alone^20^ or combined with anti-PD-1 blocking mAb, had no significant impact on progression-free survival of Neuro2a-bearing mice (Fig. 2a).

In view of the potent synergistic effects of a cell depleting anti-CD4 mAb in combination with IL-21-based immunotherapy, in the Neuro2a tumor model^20,21^, we tested the combination of anti-PD-1 and anti-CD4 mAbs according to the schedule shown in Fig. 2b (inset). This combined therapy cured 100% (5/5) of mice bearing disseminated Neuro2a-NB in two independent experiments. Notably, all mice receiving combined anti-CD4/anti-PD-1 mAb immunotherapy initially developed disseminated tumors, similarly to control mice, as evaluated by luciferase-based IVIS on day 15 after tumor challenge. However, after further 21 days tumors disappeared in most treated mice (4/5) and at day 51 from challenge all of them were completely tumor-free (Fig. 2c). As previously reported, the anti-CD4 mAb alone only marginally delayed tumor-progression of Neuro2a-luc-bearing mice (Fig. 2b).

The combination of the anti-PD-L1 blocking mAb with the cell-depleting anti-CD4 mAb had also cooperative anti-tumor effects. As shown in Fig. 2b and c, anti-PD-L1/anti-CD4 mAb combined therapy cured 4/5 mice from disseminated Neuro2a-luc tumors. Indeed, all mice showed either complete or partial tumor regression within 36 days from NB induction, although one mouse developed tumor progression at day 51. We also verified that anti-CD4 mAb produced a similar CD4^+^ T cell depletion irrespective of concomitant anti-PD-1 mAb or anti-PD-L1, treatment as detected 20 days after NB induction. In addition, CD4+ T cells were similarly depleted, in both Neuro2a- and NXS2-bearing mice (Supplementary Figure S4). The CD4 T cell counts progressively recovered until 90 days after tumor challenge, when they were almost completely reconstituted^21^.

Importantly, long-term surviving animals, which had been cured by combined anti-CD4/anti-PD-1 mAb immunotherapy, showed persistent immunity towards Luciferase-expressing, as well as parental-unmodified-tumor cells. Indeed, none of them developed progressive tumors when re-challenged with NB cells 90 days after the first NB induction. By contrast, simultaneously injected naïve mice developed rapid tumor progression (Supplementary Figure S5a). Initially also 2/5 Neuro2a-cured mice developed small tumors after re-challenge, which were, however, subsequently rejected as indicated by IVIS (Supplementary Figure S5b and Supplementary Table S1). A third challenge 90 days after the second one also failed to induce progressive Neuro2a tumors (not shown) and all mice remained tumor-free as long as 270 days after the first challenge. Similarly, mice cured by anti-PD-L1/anti-CD4 mAb combination, were resistant to a second challenge with a tumorigenic dose of NB cells (Supplementary Figure S5c).

### Delayed combined immunotherapy is effective

**Figure 3:**
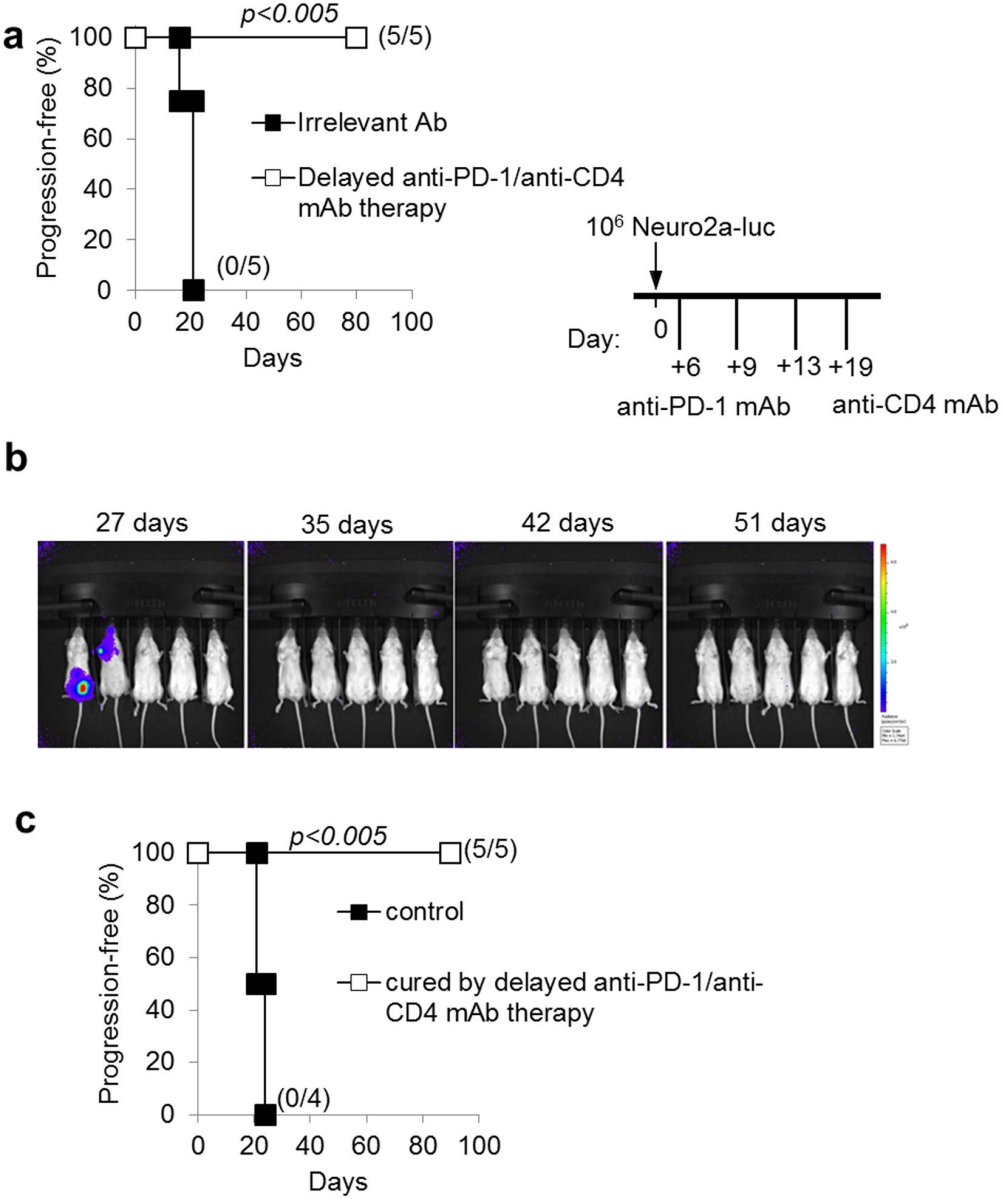
Delayed combination therapy with anti-PD-1 and anti-CD4 mAb is effective and leads to a long-lasting immunity to NB. (**a**) The combined treatment with anti-PD-1 and anti-CD4 mAb potently inhibits Neuroblastoma progression even if started at a later time point (+6 days from challenge). P values of delayed combined treatments vs irrelevant Ab controls are indicated (Wilcoxon log-rank test). Percentages of progression-free mice are indicated on the Y-axis and the fraction of progression-free mice of each group is given in brackets. (**b**) IVIS analysis representation of Neuro2a-luc NB development in mice receiving delayed combined treatments at the indicated time-points from challenge. (**c**) All mice cured by combined anti-PD-1/anti-CD4 mAb therapy were resistant to a subsequent i.v. challenge (at 90 days from primary challenge) with a fully tumorigenic dose of Neuro2a-luc cells. P values of delayed combined treatments vs irrelevant Ab controls are indicated (Wilcoxon log-rank test).

We further evaluated the efficacy of anti-CD4/anti-PD-1 combined therapy on established tumors by initiating treatment at a later time point from NB induction (6 days post challenge). Similarly to the results obtained with early-onset therapy, in the Neuro2a model, delayed combined mAb therapy cured 100% of mice from disseminated NB (Fig. 3a), in two independent experiments. All mice receiving combined mAb immunotherapy were tumor-free at 35 days from challenge, and were cured at long term (Fig. 3b). Delayed combined immunotherapy-cured mice also survived after a second challenge with tumor cells 90 days after the first NB injection (Fig. 3c) indicating the development of long-term immunity to NB antigens.

### Combination and delayed combined therapy with anti-PD-1 plus anti-CD4 mAb showed synergistic activity also in NXS2 disseminated NB model

**Figure 4:**
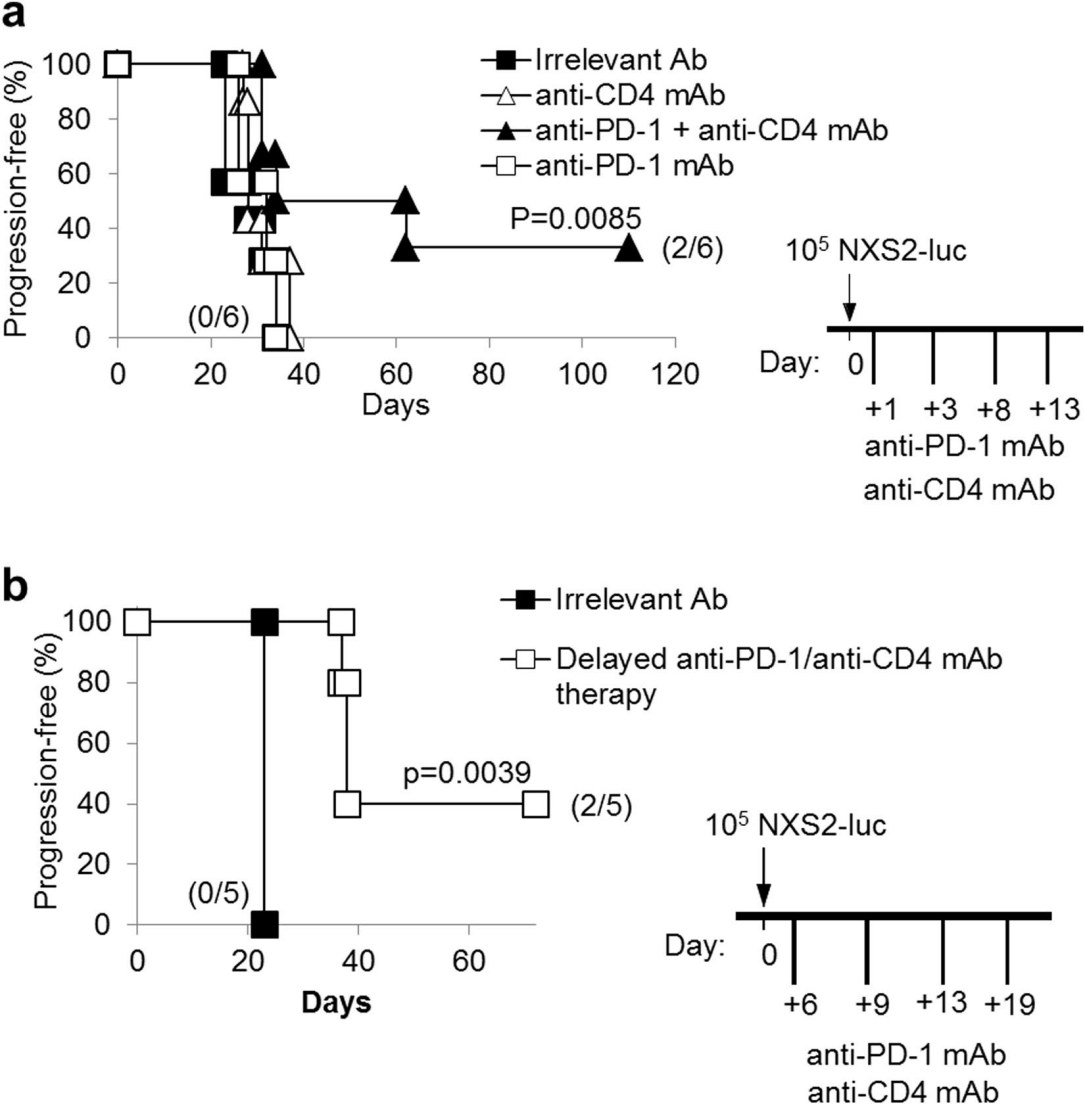
Combination therapy with anti-PD-1 and anti-CD4 mAb significantly prolong NXS2-tumor-free progression in mice. (**a**) Kaplan-Meier analysis of A/J mice challenged i.v. with a tumorigenic dose of NXS2-luc cells on day 0 and then treated with anti-PD-1 and anti-CD4 mAb shows a significant increase in mice survival, with 2/6 mice cured from disseminated tumors. P values of combined treatments vs irrelevant Ab controls are indicated (Wilcoxon log-rank test). The experiment shown is representative of two independent ones with identical results. (**b**) The combined treatment with anti-PD-1 and anti-CD4 mAb potently inhibits Neuroblastoma progression even if started at a later time point (+6 days from challenge). P values of delayed combined treatments vs irrelevant Ab controls are indicated (Wilcoxon log-rank test). Percentages of progression-free mice are indicated on the Y-axis and the fraction of progression-free mice of each group is given in brackets.

We evaluated the efficacy of combined therapy with anti-PD-1/anti-CD4 mAb in the syngeneic NXS2 NB model, which originates a very aggressive disseminated disease. The combined anti-CD4/anti-PD-1 mAb immunotherapy turned to be effective also on mice bearing the NXS2 tumor, as 33% of them remained tumor-free at long-term (Fig. 4a), in two independent experiments. Cured mice also showed resistance to re-challenge with NXS2-luc cells (data not shown). In addition, delayed-onset combined immunotherapy, started 6 days after tumor induction, significantly prolonged progression-free survival, in the NXS2 NB model (Fig. 4b).

### Mechanisms involved in anti-tumor responses induced by anti-PD-1/anti-CD4 combination immunotherapy

**Figure 5:**
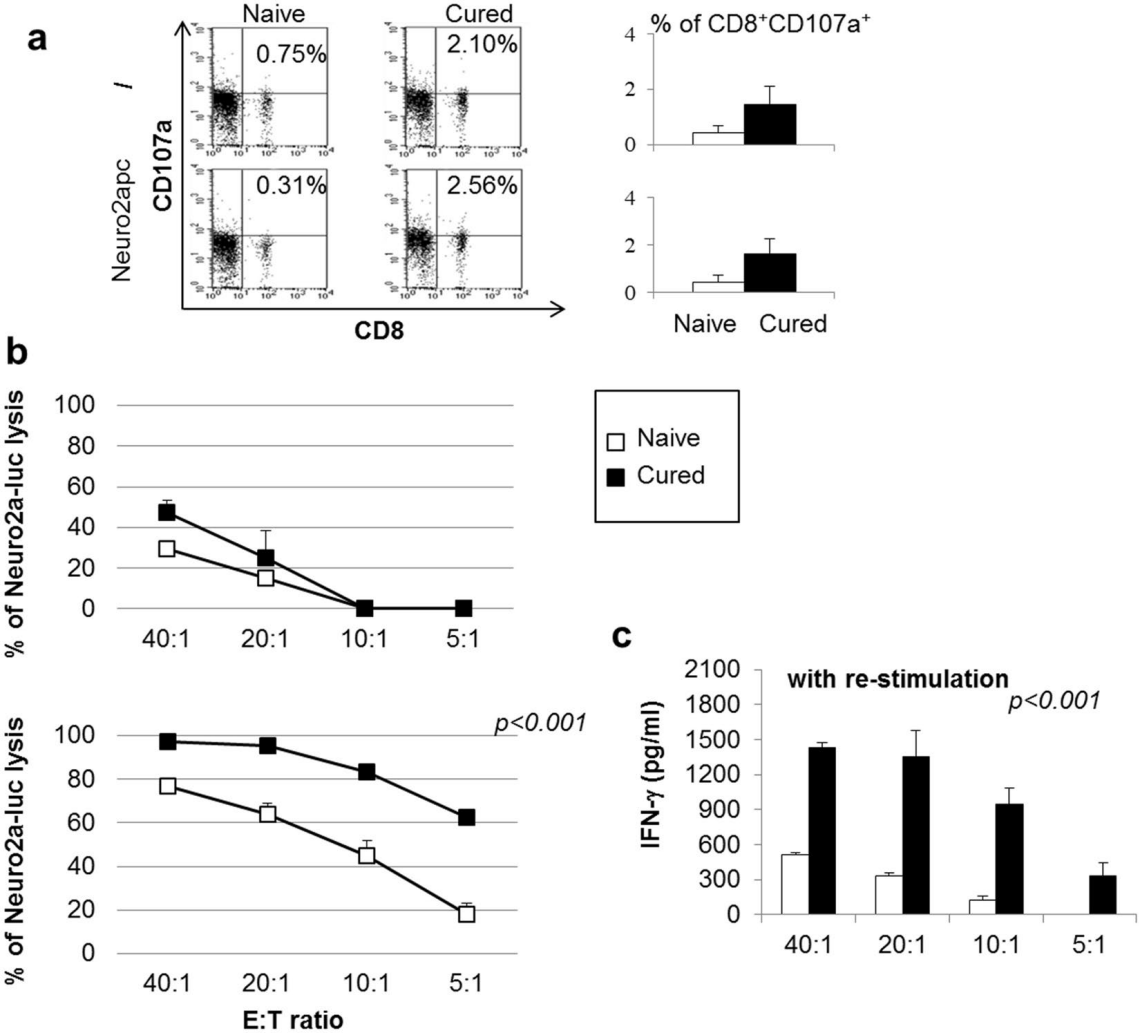
Spleen cells from mice cured by combination therapy with anti-PD-1 and anti-CD4 mAb display CTL responses *in vitro*.(**a**) Spleen cells from mice treated by combined anti-PD-1/anti-CD4 mAb therapy show higher frequency of CD8^+^CD107a^+^ CTLs relative to naïve mice. CD8^+^CD107a^+^ slightly increased after *in vitro* re-stimulation with Neuro2apc cells. Percentages of double positive cells are given. (left panel: a representative mouse is shown, right panel: M ± SD values of CD8^+^CD107a^+^ T cells from 5 mice per group are given. (**b**) Cytolytic activity of spleen cells from naïve (open boxes) and cured mice (full boxes) against Neuro2a-luc cells, at different Effector:Target (E:T) ratios (upper panel). Cytolytic activity of spleen cells from naïve (open boxes) and cured mice (full boxes), after 5-day *in vitro* MLTC stimulation with Neuro2apc cells, against Neuro2a-luc cells, at different E:T ratios (lower panel). Percentages are evaluated as indicated in the material and method section. P values are indicated (T-test). (**c**) IFN-γ production of MLTC stimulated spleen cells from naïve (open histogram) and mice cured by combined anti-PD-1/anti-CD4 mAb therapy (black histogram) after further 72 hrs stimulation with Neuro2a-luc cells, at different E:T ratios. P values are indicated (T-test).

Since anti-PD-1 therapy involves the re-activation of silenced anti-tumor CTL responses^1,26–29^, we tested whether CD8^+^ T lymphocytes were involved in the therapeutic effect obtained by combined mAb immunotherapy. To this end, we first studied the expression of CD107a, a marker of CTL de-granulation. Spleen cells from mice receiving combined anti-CD4/anti-PD-1 mAb showed an increased proportion of CD8^+^CD107a^+^ cells as compared with cells from naïve mice (Fig. 5a). The proportion of CD8^+^CD107a^+^ cells slightly increased after a 3hr-stimulation with Neuro2apc cells. These data suggested *in vivo* activation of CD8^+^ T-cell responses against NB cells in mice receiving combined immunotherapy. In addition, spleen cells from cured mice showed higher capacity to kill NB target cells as compared with cells from naïve mice in a 72 hr-cytotoxicity assay on Neuro2a-luc cells (Fig. 5b upper panel). Lymphocytes from cured or naïve mice re-stimulated *in vitro* for 5-days with irradiated Neuro2apc cells showed a more powerful cytolytic activity, since 100% of tumor cells were killed at the highest E/T ratio and lysis was >60% even at the lowest E/T ratio tested (Fig. 5b lower panel). Accordingly, spleen cells from cured mice displayed higher production of IFN-γ than those from naïve mice, in response to Neuro2apc re-stimulation (Fig. 5c).

**Figure 6:**
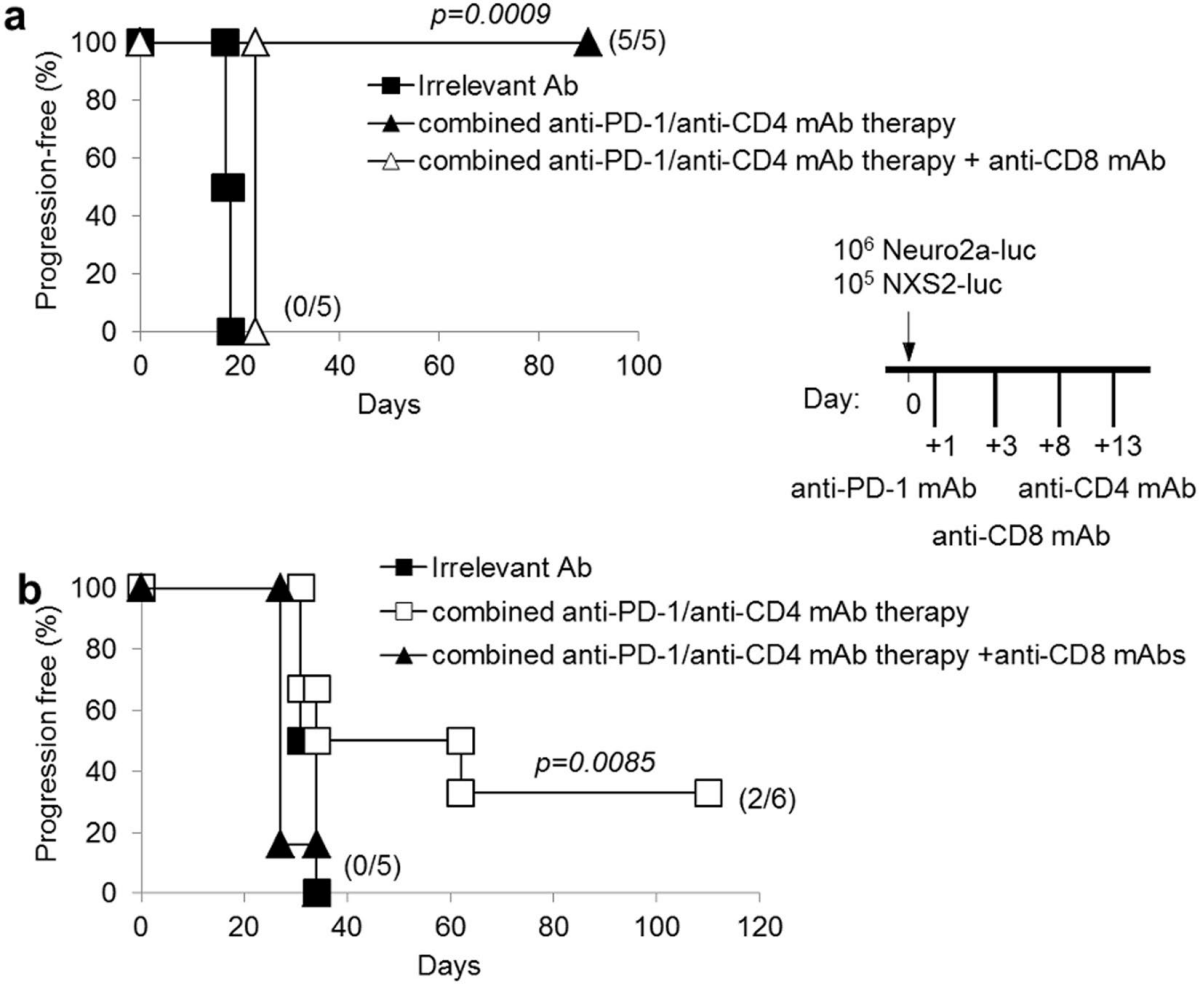
CD8^+^ T cells are the main effector cells involved in combination therapy with anti-PD-1 and anti-CD4 mAb. All mice receiving a cell-depleting anti-CD8 mAb in association with combined anti-PD-1/anti-CD4 mAb therapy, after i.v. challenge with Neuro2a-luc (panel a) or NXS2-luc (panel b) cells, develop tumors in a similar fashion as mice receiving no therapy but only irrelevant mAb. P values of combined anti-PD-1/anti-CD4 mAb therapy + anti-CD8 mAb vs combined therapy are indicated (Wilcoxon log-rank test). Percentages of progression-free mice are indicated on the Y-axis and the fraction of progression-free mice of each group is given in brackets. The schedule shown in the inset shows the timing of treatments.

Finally, an anti-CD8 depleting mAb was administered to tumor-bearing mice together with combined anti-CD4/anti-PD-1 mAb therapy. Anti-CD8 mAb treatment completely abrogated the therapeutic effects of combined immunotherapy in both Neuro2a and NXS2 NB models (Fig. 6). Altogether, these data demonstrate that combined anti-CD4/anti-PD-1 mAb immunotherapy induces tumor rejection through a CD8^+^ CTL response.

To assess the possible involvement of B-cell responses in combined mAb treatment, we screened the sera from cured mice for surface reactivity with viable Neuro2a-luc/NXS2-luc or Neuro2apc/NXS2pc cells, using an anti-total Ig secondary anti-serum. However, no significant antibody reactivity against NB cells was detected at any dilution tested (Supplementary Figure S6).

In the attempt to explain the different efficacy generated by combined immunotherapy in the two syngeneic models of NB, we studied the tumor microenvironment (TME) in both Neuro2a and NXS2 pseudo-metastatic tumors. Haematoxylin/eosin staining showed more abundant blood vessels in Neuro2a tumors than in NXS2 tumors (Supplementary Figure S7). Immunohistochemistry analysis of paraffin embedded tumor sections showed more abundant CD3^+^ and CD4^+^ T cells infiltrating NXS2, than Neuro2a tumors, while B220^+^ B cells and myeloid cells (identified by staining for myeloperoxidase) were similarly represented (Supplementary Figure S7).

Immunofluorescence and FACS analyses, suggested the presence of more abundant CD4^+^CD25^−^Lag3^+^ Tr-1 cells in NXS2 than in Neuro2a tumors, while CD4^+^CD25^+^ Treg cell counts were similar in both tumor types (Supplementary Figure S8).

To further study possible differences among the two types of tumors, we collected tumors from mice whose tumors seemed not to reduce their ROI signal at IVIS analysis 7 days after the end of treatments. Tumors developed in NXS2-bearing mice displayed a more abundant CD11b^+^Gr-1^+^ myeloid derived suppressor cells (MDSC) infiltrate, in respect to Neuro2a-tumors (Supplementary Figure S9), suggestive of a potential role for these cells in NXS2-associated immune suppression, which could limit the effect of combined immunotherapy.

## Discussion

In this study, we show that murine NB cells express PD-L1 molecule, but, differently from other preclinical tumor models^25,27^, the use of anti-PD-1 or anti-PD-L1 blocking mAb alone did not inhibit progression of NB in syngeneic mice. However, a combination therapy of anti-PD-1 or anti-PD-L1 mAb with an anti-CD4 mAb showed a synergistic effect, in two syngeneic NB models.

Dysregulation of immune checkpoints is considered one of the principal mechanisms through which tumors escape immune system recognition and undergo progression. For example, expression of PD-L1 on mouse tumor cells increases apoptosis of tumor-reactive CTLs, inhibits their cytotoxic activity *in vitro* and promotes tumor growth *in vivo* ^27,30,31^. Studies in murine models demonstrated that blockade of PD-L1 results in immune-mediated destruction of different PD-L1-expressing tumors, including myeloma, melanoma, and mammary carcinoma^24,31^. The lack of effects of anti-PD-L1 or anti-PD-1 mAb mono-therapy in our NB model does not seem related to differences in the type or dosage of mAb used, since the mAb clone and the doses are the same reported to be successful in other experimental models^31^. This observation rather suggests that different immune-regulatory mechanisms, independent of the PD-L1/PD-1 checkpoint, may have a relevant role in the present NB models. It is well known that the conversion of Treg cells from precursors and/or their expansion within the tumor microenvironment may represent an important mechanism of immune evasion^32^. Indeed, our previous results indicated that CD4^+^CD25^+^FoxP3^+^ Treg cells increase in the lymphoid organs of mice during Neuro2a NB development^20^. We therefore tried to combine PD-1 blockade with either an inhibitor of NTPDase, POM-1, or an anti-CD25 mAb targeting CD25^+^ Treg cells, which was previously shown to cooperate with IL-21-based immunotherapy in other syngeneic models^33^. However, also these combined treatments showed no substantial effect on NB progression. We can conclude that if Treg cells are involved in NB-induced immune suppression, adenosine generation is not the predominant mechanism involved. On the other hand, it has to be considered that treatment with anti-CD25 mAb efficiently depleted CD4^+^CD25^high^ Treg cells, in the syngeneic NB model, but a population of CD4^+^CD25^−/low^FoxP3^+^ cells or other CD4^+^ Treg precursors were left untouched^20^. In addition, other subsets of CD4^+^CD25^−^ T cells display immune-regulatory functions, e.g. Tr1 cells^32^, which would escape from CD25 mAb control. Indeed, the use of an anti-CD4 mAb transiently depleting total CD4^+^ T cells was more effective than anti-CD25 mAb when used in combination with the Th-derived cytokine IL-21 in our previous reports^20,21^. In these previous studies, we hypothesized that IL-21 could sustain CTL responses in the absence of CD4^+^ Th cells.

Here, we show that the combination of the anti-CD4 depleting mAb with either PD-1 or PD-L1-blocking mAbs has anti-tumor synergistic effects, leading to 100% and 90% cure rates, in the Neuro2a model, respectively. These are the best results reported so far with immunotherapy in this disseminated NB. In addition, the combined anti-CD4/anti-PD-1 mAb immunotherapy showed efficacy also on mice bearing NXS2 tumors. Of note, mice that rejected NB after combined immunotherapy, showed long-term remissions and immunity to NB antigens without signs of toxicities or immunological disorders. Immunity to NB antigens was demonstrated by re-challenge with a fully tumorigenic dose of NB cells, in mice cured by combined treatment at 90 days from the initial NB induction. At this time point the CD4^+^ T cell population was completely reconstituted^20^. It is likely that the reconstituted population switches from immune-regulatory to anti-tumor helper functions and thus participates in sustaining anti-NB effector CD8^+^ T cell responses during re-challenge. Indeed, in previous studies of NB immunotherapy by anti-CD4+ IL-21, the reconstituted CD4^+^ T cells were fundamental for rejection of a second tumorigenic challenge of Neuro2a cells^21^.

Importantly, the combined immunotherapy showed a similar efficacy in both Neuro2a and NXS2 models, even if started at later time points when tumor burden in mice was higher.

CD8^+^ CTLs were essential anti-tumor effector cells in mice treated with the anti-CD4/anti-PD-1 mAb combination as CD8^+^ T cell depletion *in vivo* completely abrogated the effect of the combined immunotherapy. This observation raises the question on how the CD8^+^ T cell response could occur in the absence of Th-derived cytokines. It is conceivable that the expansion of CD8^+^ T cells responding to NB antigens may be initially supported by endogenous cytokines produced by a residual Th cell population, as full CD4^+^ T cell depletion required four subsequent anti-CD4 mAb injections. During CD4^+^ cell depletion, CD8^+^ T cells may be supported by a homeostatic cytokine response^20,21,34^ necessary for replenishment of the CD4^+^ population, which is completed within 90 days^20^. Also, CD8^+^ CTLs are kept active during NB regression by removal of PD-1 inhibition and can thus exert their anti-tumor activity. Our present findings support the concept that, at least in these NB models, CD4^+^ T cell-related immune-suppressive mechanisms play an essential role, as indicated by the need of simultaneous usage of anti-CD4 mAb and PD1/PD-L1 blockade to induce long-lasting remissions. However, it is possible that PD1^+^ CD4^+^ T cells may compete for anti PD-1 mAb binding to CD8^+^ or tumor cells, respectively. Therefore, we cannot exclude that the anti-CD4 mAb may potentiate the effect of anti-PD-1 mAbs also through removal of competing cells. Indeed, the proportions of CD4^+^PD1^+^ T cells increased up to two-fold, while CD8^+^PD-1^+^ cells showed no significant changes, in the lymph nodes of NB-bearing, relative to naïve mice (Supplementary Figure S10). In view of the greater efficacy of combined immunotherapy in the Neuro2a model, we explored possible differences in the TME of Neuro2a and NXS2-tumors. Somehow surprisingly, immunohistochemistry indicated more abundant CD3^+^ and to a lesser extent CD4^+^ T cell infiltrates in NXS2 than in Neuro2a pseudo-metastases. The gap between CD3^+^ and CD4^+^ T cells infiltrating NXS2 tumors (Supplementary Figure S7) may suggest the presence of a CD3^+^CD4^−^ cell population endowed with immune-regulatory functions, which will be addressed in further studies. In addition, FACS analyses suggested more abundant CD4^+^ CD25^−^Lag3^+^ Tr-1 cells, in NXS2 tumors while CD4^+^CD25^+^ Treg cells were similarly represented in the two NB models. Although we found no evident differences in the CD11b^+^GR1^+^ population among Neuro2a and NXS2 tumors, in untreated mice, these cells appeared to be more expanded in NXS2 than in Neuro2a tumors from mice receiving combined immunotherapy. We may speculate that the post-therapy expansion of a CD11b^+^GR1^+^ MDSC population may represent an additional immune-regulatory mechanism, which may explain the lower efficacy of combined immunotherapy, in the NXS2 NB model. A note of caution is that we don’t know whether the NXS2 tumors studied would have been eventually rejected, although we chose nodules that showed no decrease in their ROI value upon IVIS examination. In any case, the potential role of MDSC will be the object of further studies in the NXS2 model.

A synergistic activity of anti-CD4 and anti-PD-1/PD-L1 mAbs has also been demonstrated in a murine model of chronic lymphocytic choriomeningitis, where PD-L1 blockade in association with CD4^+^ T-cell depletion successfully rescued exhausted CD8^+^ T cells and boosted antiviral control^35^. Another study showed that combined anti-CD4+ anti-PD-1/PD-L1 mAb therapy synergistically mediated B16-F10 melanoma or C26 colon cancer rejection, in syngeneic mice^36^. In a syngeneic murine model of mesothelioma anti-CD4+ anti-PD-1/PD-L1 mAb initially induced tumor regression, followed by an accelerated tumor growth concomitant to CD4 T cell recovery^37^. However, opposite results have been recently published in other syngeneic tumor models using the carcinogen-induced MC38 adenocarcinoma or the YUMM1.1 and YUMM2.1 melanoma cell lines. In these tumor models, CD4^+^ T cells were strictly necessary for the anti-tumor effects of anti-PD-1 mAb therapy as their depletion abrogated the therapeutic activity^31^.

In patients with NB, mAbs targeting PD-L1 (NCT02541604) or PD-1 (NCT02332668, NCT02304458, NCT02914405, NCT 01822652) are currently being tested. It may be possible that anti-PD-1 or anti-PD-L1 mAb used as single agents have limited impact in human NB, which displays a low mutational load^38,39^, although this increase after recurrence^40^. Indeed, there is a correlation between response rate to immune-checkpoint blockers and the levels of somatic non-synonymous mutations, which potentially generate neo-antigens in tumors^38,39^. Therefore, it is conceivable that combinations of different immune checkpoint blockers might be more effective in NB, and studies combining anti-PD-1 with anti-CTLA4 mAb have been planned (NCT02304458).

It has been proposed that the limited efficacy of anti-PD-1 therapy, in adult patients, might be explained by the development of an ‘adaptive resistance’ to blocking mAbs involving the up-regulation of alternative immune checkpoints such as TIM-3^41^. However, in the Neuro2a model, combination of anti-PD1 and anti-TIM-3 mAb therapy did not result in an increase in progression free survival of mice, indicating that TIM-3 immune checkpoint was not implicated in Neuro2a immune escape (Supplementary Figure S11).

In view of the promising experimental results of combined anti-CD4+ anti-PD-1/PD-L1 mAbs therapy in our syngeneic model of disseminated NB and of previous reports of anti-CD4 mAbs for clinical use in humans^42,43^, this treatment may be potentially translated to NB patients. Indeed, anti-CD4 mAb treatment showed activity in CD4^+^ T cell lymphomas, without significantly increasing the risk of life-threatening infections^42,43^. However, the role of CD4^+^ immune regulatory T cell subsets is still debated in NB patients and controversial data on the potential role of Treg have been reported^44–46^, while CD4^+^LAG-3^+^ Tr1 cells seemed reduced^46^. Finally, further studies in pre-clinical models should be considered to confirm the possible efficacy of this novel therapeutic strategy before clinical testing in patients with refractory or relapsing NB.

## Materials and Methods

### Cell cultures and IFN-γ treatment

Neuro2a-luc and NXS2-luc obtained by transducing Neuro2a (ATL99007, ICLC, Genoa, Italy; authentication by institutional biological banking facility using STR according to International Cell Line Authentication Committee (ICLAC) guidelines) or NXS2^47^ parental cells (pc) were grown in DMEM medium as described^48^. For retroviral plasmid production, the firefly luciferase gene luc2 (Photinus pyralis) was obtained as a XhoI/XbaI fragment from pGL4.10 vector (Promega) and cloned into the LXIN retroviral vector (Clontech) to obtain L-LUC2-IN. Retrovirus was obtained as described^49^ and used to infect cells for the constitutive expression of the luciferase gene. Neuro2apc and NXS2pc cells were treated with Rat IFN-γ (1,000 IU/ml; Peprotech) for 48 hrs. Cell lines are routinely tested for *Mycoplasma* infections by PCR analysis and immunofluorescence analysis prior to *in vivo* injections.

### Animal models and treatments

Five week-old female A/J mice were purchased from ENVIGO (Udine, Italy). The animals were housed in pathogen-free colony, and experiments were performed in accordance to the National Regulation on Animal Research Resources and approved by the Review Board of the IRCCS AOU San Martino-IST, Genoa, and Italian Ministry of Health (n° 358, Dl.vo 116/92, and 412, Dl.vo 4/03/2016 n 26). Mice were injected intra venous (i.v.) in the tail vein with Neuro2a-luc or NXS2-luc (>90% viable; 1 × 10^6^ cells or 1 × 10^5^ in a volume of 100 μl of serum-free medium, respectively) and were randomly separated in groups of 5–10 animals/group. Murine recombinant (r)IL-21 (1 μg/mouse) was given by subcutaneous (s.c.) injections at days +2, +6, +9, +12, +16 post i.v. challenge. Anti-CD4 (GK 1.5) (ATCC, Rockville, MD) at 100 μg/mouse/dose^21^ or anti-PD-1 (RMP1-14)^31^, anti-PD-L1 (10 F.9G2)^31^ and anti-TIM-3 (RMT3-23) mAb^50^ (BioXCell, West Lebanon, NH, USA), each at 200 μ μg/mouse/dose, were given intraperitoneally (i.p.) at days +1, +3, +8, +13. For delayed combined immunotherapy, anti-PD-1 or anti-CD4 were administrated i.p. at days +6, +9, +13, +19 from IV challenge. Depletion studies were performed by i.p. injection of anti-CD8 (2.43) and anti-CD25 (pc61) blocking mAb as reported^20^. *In vivo* irrelevant Ig control was anti-KLH LTF2 (BioXCell, 200 μg/mouse/dose). The NTPDase inhibitor sodium polyoxotungstate (POM-1) was purchased from Santa Cruz, solubilized in PBS, and given to mice i.p. daily starting from the day after the i.v. challenge for 10 days, at the dose of 5 mg/kg.

In general, mice were monitored for disease symptoms every other day (starting from two weeks after tumor challenge) manually and weekly by IVIS inspection, and were sacrificed by CO_2_ asphyxiation when they showed a ROI signal equal to or over 9 × 10^9^ photons/seconds/cm^2^/sr or weight loss (>15%) or presence of tumor masses or any other sign of disease. All the *in vivo* experiments presented are representative of the general result, and were performed at least twice, with similar results.

### Analysis by *In Vivo* Imaging Systems (IVIS)

Mice were treated with an i.p. injection of the substrate luciferin (150 mg/Kg), 5 to 10 minutes before photons acquisition. The mice were anesthetized by inhalation of isoflurane (Induction phase: 1–3% in O_2_, Mantenance phase: 2% in O_2_) continuously dispensed for the duration of the procedure. The mouse were placed under anesthesia in the imaging chamber and the bioluminescence images were acquired immediately and commuted in digital signals expressed as photons/seconds/cm^2^/sr. Quantification of the *in vivo* Luciferase signal of the tumor area, was made by defining an area of photon emission measurement (Region Of Interest-ROI) for each animal analyzed using Living Image software (Xenogen Corporation-Perkin Elmer, USA). The ROI signal expressed as fold increase of photons over time, was computed at the days indicated in each figure, with respect to the starting day of injection of tumor cells (T0). A ROI signal equal to or over 9 × 10^9^ photons/seconds/cm^2^/sr will be considered as an indicator of tumor progression in the absence of any sign of suffering.

### RNA and RT-PCR analysis

Total RNA was extracted from Neuro2apc or NXS2pc cells (1 × 10^6^) using the RNeasy kit (Qiagen, Cologne, Germany). One μg of total RNA was then reverse-transcribed and 5 μl of cDNA was amplified, in a final volume of 50 μl, with 2.5 IU FastStart Taq DNA Polymerase (Roche), using primers specific for the housekeeping gene β-actin (upper: ggCATCgTgATggACTCCg, lower: gCTggAAggTggACAgCgA) and PD-L1 (upper: TgCTgCATAATCAgCTACgg, lower: gCTggTCACATTgAgAAgCA). The amplification products were then analyzed on 2% agarose gel.

### Immunofluorescence and FACS analysis

Splenocytes or lymphocytes were stained using anti-PD-L1 PE, anti-PD-1 PE, anti-CD4 FITC, anti-CD8-FITC and anti-CD107a-PE (eBioscience, San Diego, CA) as indicated by the manufacturer. Specific mAb isotype-matched control was also used. Sera from naïve and mice cured by combined anti-PD-1 and anti-CD4 mAb therapy were used to assess reactivity against Neuro2apc or NXS2pc cells at dilutions ranging from 1:10 to 1:10,000. A FITC-conjugated goat anti-mouse antiserum (Jackson Labs,West Grove, PA) was used as a second-step reagent. Anti-GD2 mAb supernatant (M36.1-S2a) (ATCC) was used as positive control.

Tumors from Neuro2a- and NXS2 bearing mice were dissociated mechanically and immune cells infiltrates were stained with anti-CD4 FITC, anti-CD25 APC, anti-LAG-3 PE, anti-Gr1 FITC and anti-CD11b PE (eBioscience, San Diego, CA) as indicated by the manufacturer. Samples were analyzed by a FACScan or FACSCalibur analyzer (Becton Dickinson).

### *In vitro* re-stimulation and cytotoxicity

Spleen cells harvested from combined mAb therapy cured or naïve mice were re-stimulated *in vitro* in a 5-day co-culture experiment, alone or with irradiated Neuro2a cells (250 Gy, by IBL 437C (CIS Bio International)), in the presence of low concentrations of rhIL-2. On the fifth day of culture, spleen cells were seeded in triplicate onto 96 well optical plates (Thermo Scientific) at serial 1:2 dilutions (from 40:1 to 5:1, Effector:Target) with Neuro2a-Luc; after another 2 days, incubation supernatant was taken for IFN-γ ELISA detection and seeded cells were used to evaluate the amount of bioluminescence produced by alive Neuro2a-Luc cells and measured by a Luminometer (Mithras LB 940 Multimode Microplate Reader). Percent of lysis was calculated from the average of triplicate well counts with the following equation: % specific lysis = 100x(spontaneous death Relative Light Units (RLU) – test RLU)/(spontaneous death RLU – maximal killing RLU)^51^.

### IFN-γ ELISA in co-culture supernatant

Supernatant from citotoxicity experiments were tested for IFN-γ using specific Duo set ELISA kit (Bender MedSystems GmbH, Vienna, Austria) following the manufacturer’s instructions.

### Immunohistochemistry analysis

Tumour samples were collected, fixed 24 hour in 4% neutral buffered formalin and embedded in paraffin, 4 μm thick sections were dewaxed and hydrated for haematoxylin/eosin or immunohistochemistry (IHC) staining. For IHC we used the following antibodies: Rabbit monoclonal anti CD3, 1:150 (Abcam, Cambridge, UK, EU); Rabbit polyclonal anti myeloperoxidase (MPO), 1:300 (Agilent/Dako, Santa Clara, CA USA); Biotinylated Rat monoclonal to CD4, 10 μg/ml (eBioscience - Thermo Fisher, Waltham, MA USA); Rat monoclonal anti B220 (CD45R) 1:200 (R&D System, Minneapolis, MN USA). Briefly, after rehydration tissue sections were boiled in citrate buffer, pH 6, for antigen retrieval, block of endogenous peroxidases was obtained in 4% H_2_O_2_, while unspecific sites were saturated with 10% normal goat serum. Primary antibodies were incubated 1 hr at RT followed by washing in TBS (0.05M TRIS-HCl pH 7.6; 0.15M NaCl; 0.05% Tween20) and 30 min incubation with the appropriate biotinylated secondary antibody (BioSPA, Milan, IT, EU). After washing and incubation with hoseradish peroxidase (HRP)-conjugated streptavidin (BioSPA), the reaction was evidenced with DAB (Vector Laboratories, Burligame, CA, USA). Finally the sections were counterstained with haematoxylin and mounted in Eukitt (Bio-Optica, Milan, IT, EU).

Cell counting was performed on randomly taken photographs of IHC-stained sections from four independent samples, using an oil-immersion 100x objective. Ten photos were acquired from each slide.

### Statistical analyses

Progression-free survival curves were constructed using the Kaplan-Meier method and the generalized Wilcoxon log-rank test was used to statistically compare the curves. Mean progression-free survival times were calculated with 95% confidence interval. The comparison between the cytotoxic activity or IFN-γ production in the supernatant from naïve or cured mice spleen cells was evaluated using T-test for independent samples. All tests were two sided. P values lower than 0.05 were considered as significant. Statistical analyses were performed using the Prism 3.0 software (Microsoft Inc).

## Electronic supplementary material

Supplementary File
