# Supplementary material for: Combined immunotherapy with anti-PDL-1/PD-1 and anti-CD4 antibodies cures syngeneic disseminated neuroblastoma
Source: Sci Rep. 2017 Oct 25;7:14049. doi: 10.1038/s41598-017-14417-6 (PMC5656588; doi:10.1038/s41598-017-14417-6)
Supplement: Supplementary file 1 — Supplementary File [file 41598_2017_14417_MOESM1_ESM.pdf]

Combined immunotherapy with anti-PDL-1/PD-1 and anti-CD4 antibodies cures syngeneic disseminated neuroblastoma

Valentina Rigo[1], Laura Emionite[2], Antonio Daga[1], Simonetta Astigiano[1], Maria Valeria Corrias[3], Concetta Quintarelli[4,5], Franco Locatelli[4,6], Silvano Ferrini[1§] and Michela Croce[1§*].

[1] Dipartimento di terapie oncologiche integrate, [2] Dipartimento della diagnostica, della patologia e delle cure ad alta complessità tecnologica, IRCCS A. O. U. San Martino-IST, Istituto Nazionale per la Ricerca sul Cancro, Largo R. Benzi 10, 16132, Genoa, Italy; [3] Dipartimento Ricerca Traslazionale, Medicina di Laboratorio, Diagnostica e Servizi, IRCCS Gaslini Institute, L.go G. Gaslini 5, 16147, Genoa, Italy; [4] Dipartimento di Oncoematologia Pediatrica, IRCCS Ospedale Pediatrico Bambino Gesù, Roma; [5] Dipartimento di Medicina Clinica e Chirurgia, Università di Napoli Federico II; [6] Dipartimento di Scienze Pediatriche, Università di Pavia.

Supplementary Figure S1

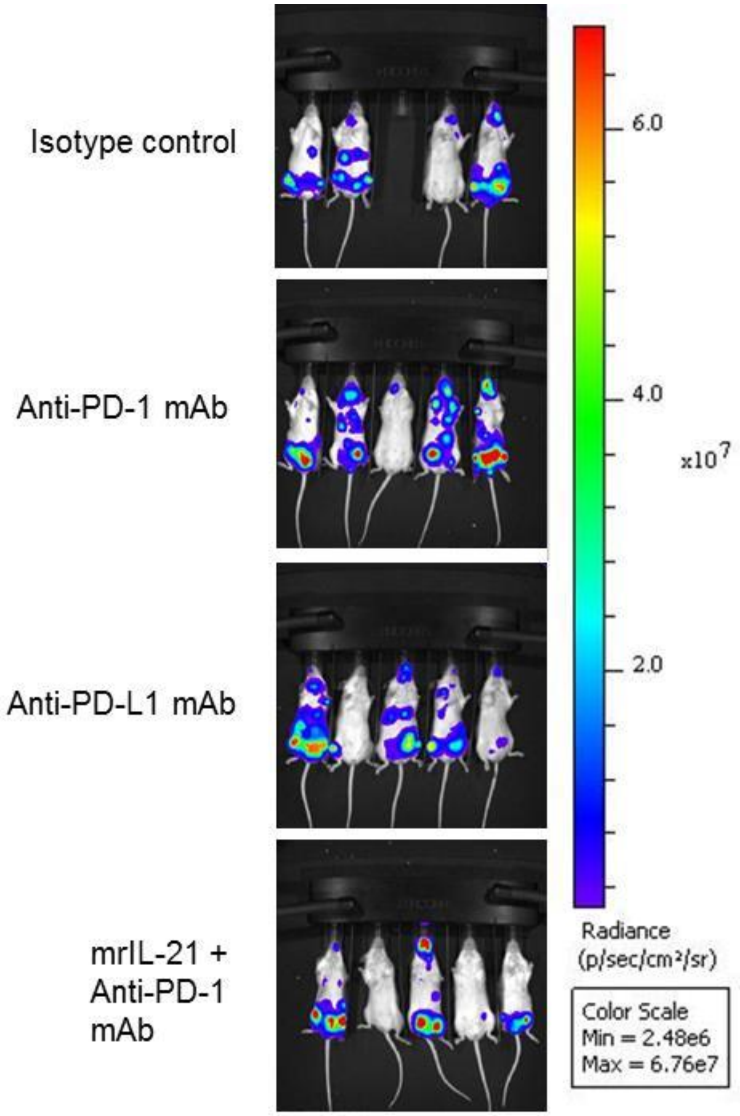

Supplementary Figure S1: IVIS analysis of Neuro2a-luc NB development in mice receiving an irrelevant Ab, anti-PD-1, anti-PD-L1 or anti-PD-1 mAb in association with rIL-21 at 15 days from challenge. Schedule of treatments are the same as those present in the inset of Figure 1c.

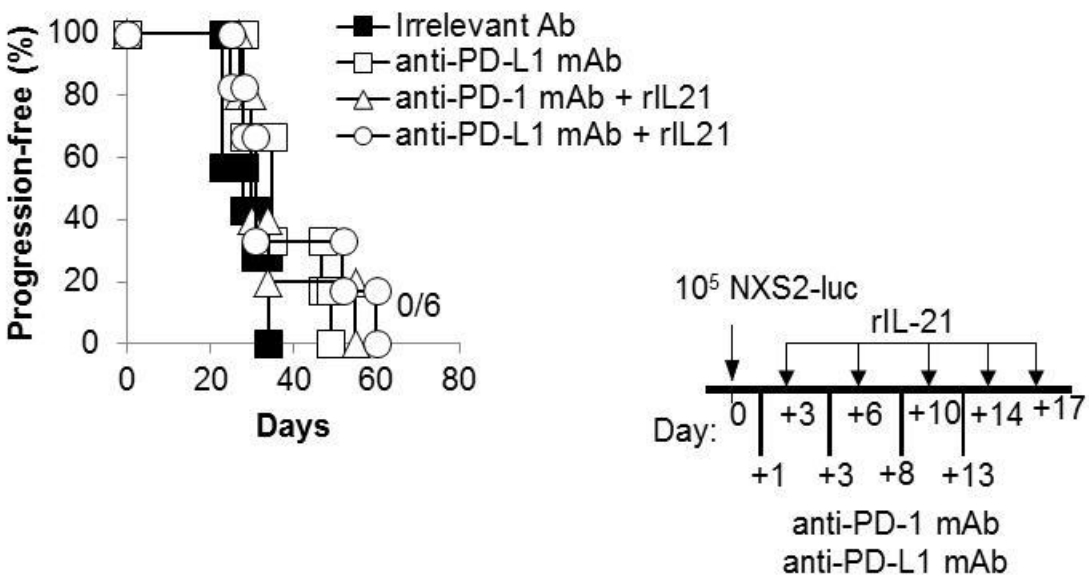

Supplementary Figure S2: Kaplan-Meier analysis of A/J mice inoculated i.v. with a tumorigenic dose of NXS2-luc cells on day 0 and treated with an irrelevant antibody, anti-PD-L1, anti-PD-L1 combined with rIL-21, or with anti-PD-1 combined with rIL-21, according to the schedule shown in the inset. Percentages of progression-free mice are indicated on the Y-axis and the fraction of progression-free mice of each group is given in brackets.

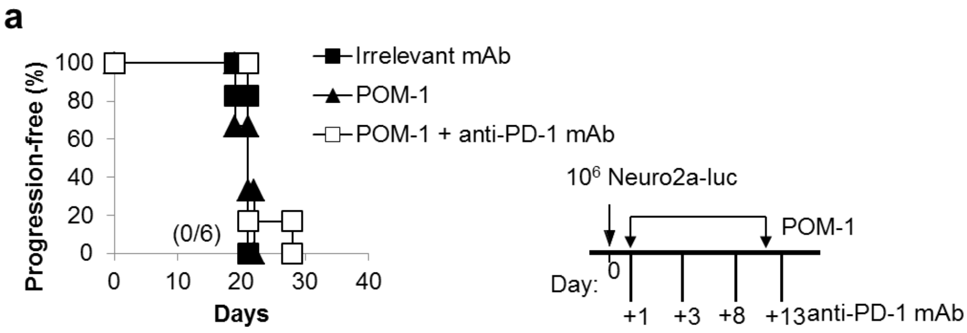

Supplementary Figure S3: a: Kaplan-Meier analysis of A/J mice challenged i.v. with a tumorigenic dose of Neuro2a-luc cells on day 0 and then treated with POM-1 alone or combined with anti-PD-1 mAb or with an irrelevant antibody, according to the schedule shown in the inset. Percentages of progression-free mice are indicated on the Y-axis and the fraction of progression-free mice of each group is given in brackets.

Supplementary Figure S4

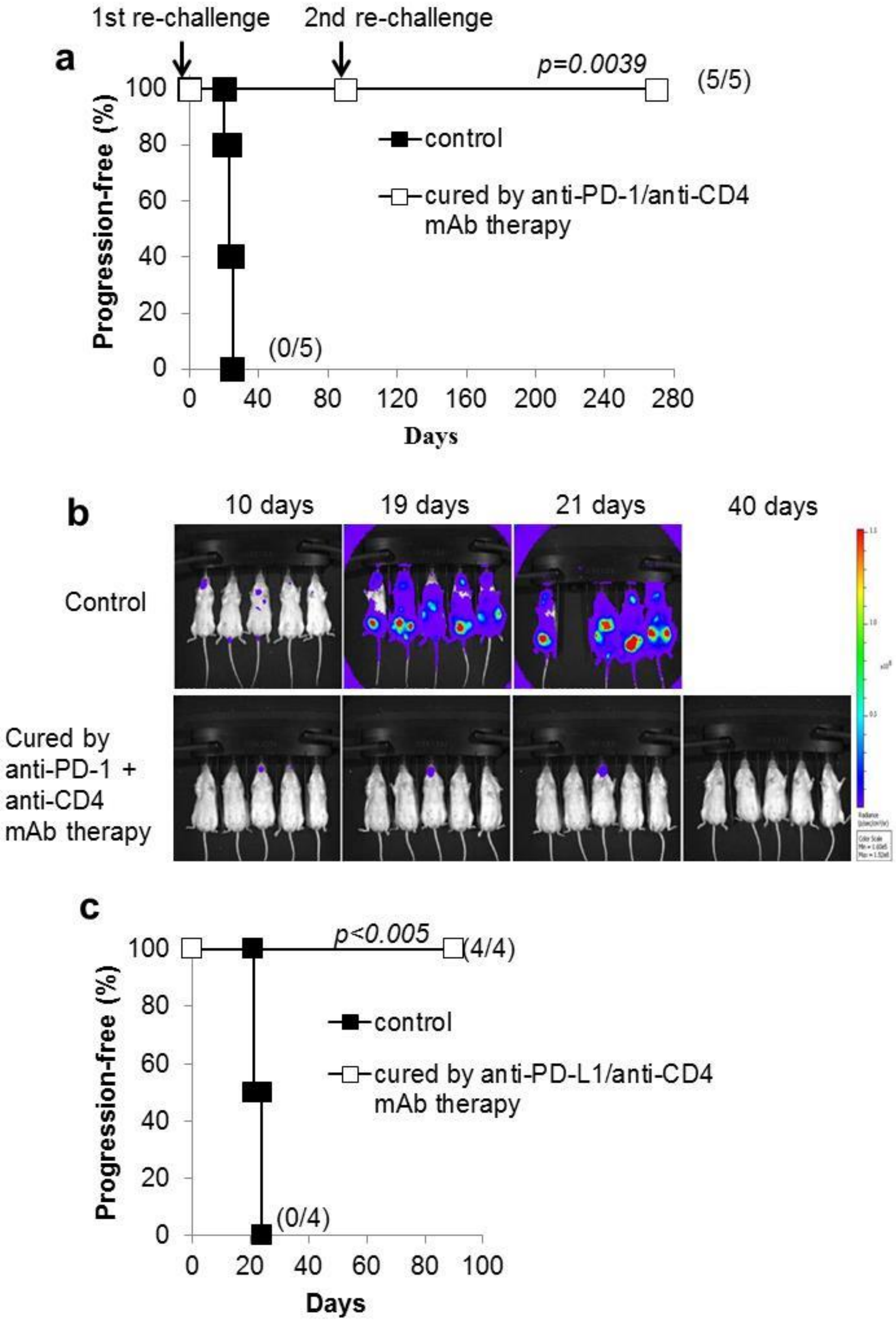

Supplementary Figure S4: a: All mice cured by combined anti-PD-1/anti-CD4 mAb therapy were resistant to subsequent i.v. challenges (at 90 and 180 days from primary challenge) with a fully tumorigenic dose of Neuro2a-luc cells. The experiment shown is representative of two independent ones with identical results. P values are indicated (Wilcoxon log-rank test). Percentages of progression-free mice are indicated on the Y-axis and the fraction of progression-free mice of each group is given in brackets. **b:** IVIS analysis of Neuro2a-luc NB development in immunotherapy cured vs. naïve mice at the indicated time-points from re-challenge. **c:** Mice cured by combined anti-PD-L1/anti-CD4 mAb therapy were resistant to a subsequent i.v. challenge (at 90 days from primary challenge) with Neuro2a-luc cells. P values are indicated (Wilcoxon log-rank test). Percentages of progression-free mice are indicated on the Y-axis and the fraction of progression-free mice of each group is given in brackets.

Supplementary Figure S5

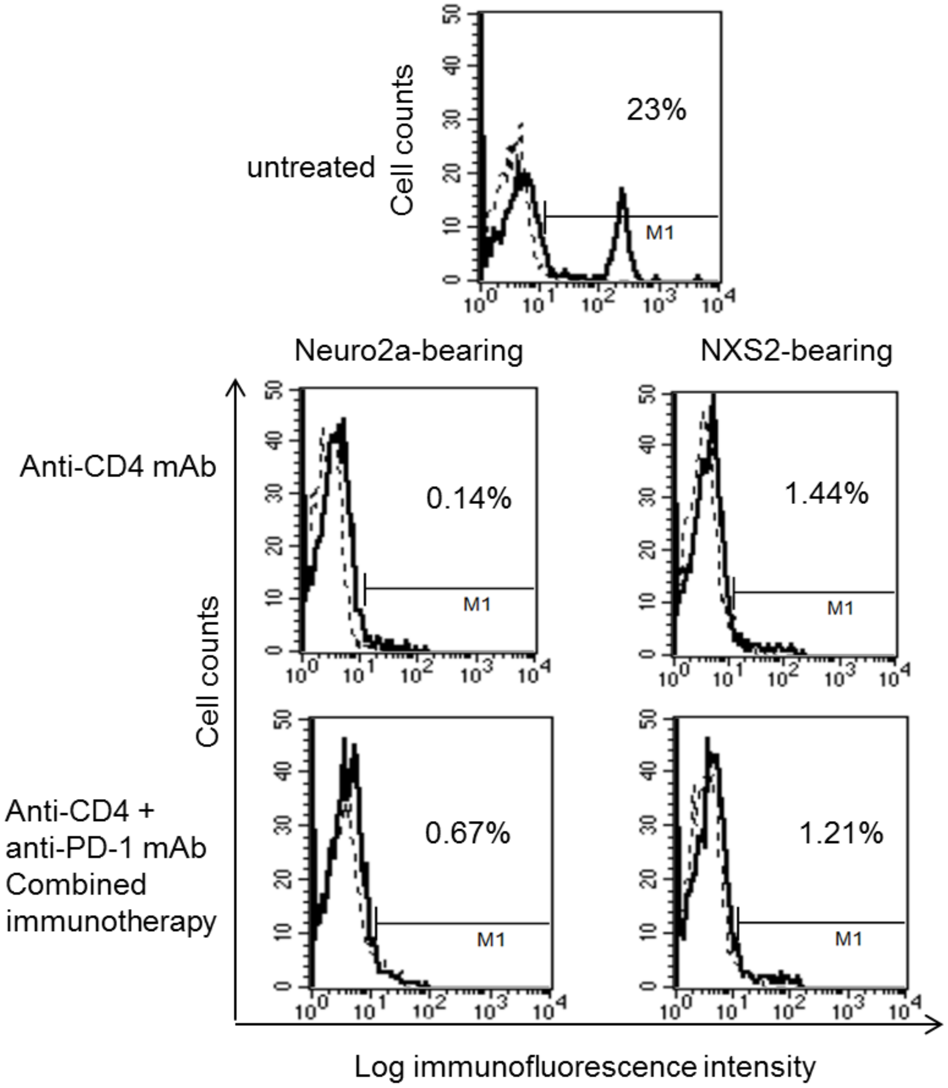

Supplementary Figure S5: FACS analysis of $CD4^+$ T cells in the spleen cells from untreated (high) and Neuro2a-, NXS2-bearing mice after anti-CD4 mAb treatment alone (middle) or anti-CD4 + anti-PD-1 mAb combined immunotherapy (bottom) at 7 days after the end of treatments. The percentage of CD4+ T cells is indicated in each histogram.

Supplementary Figure S6

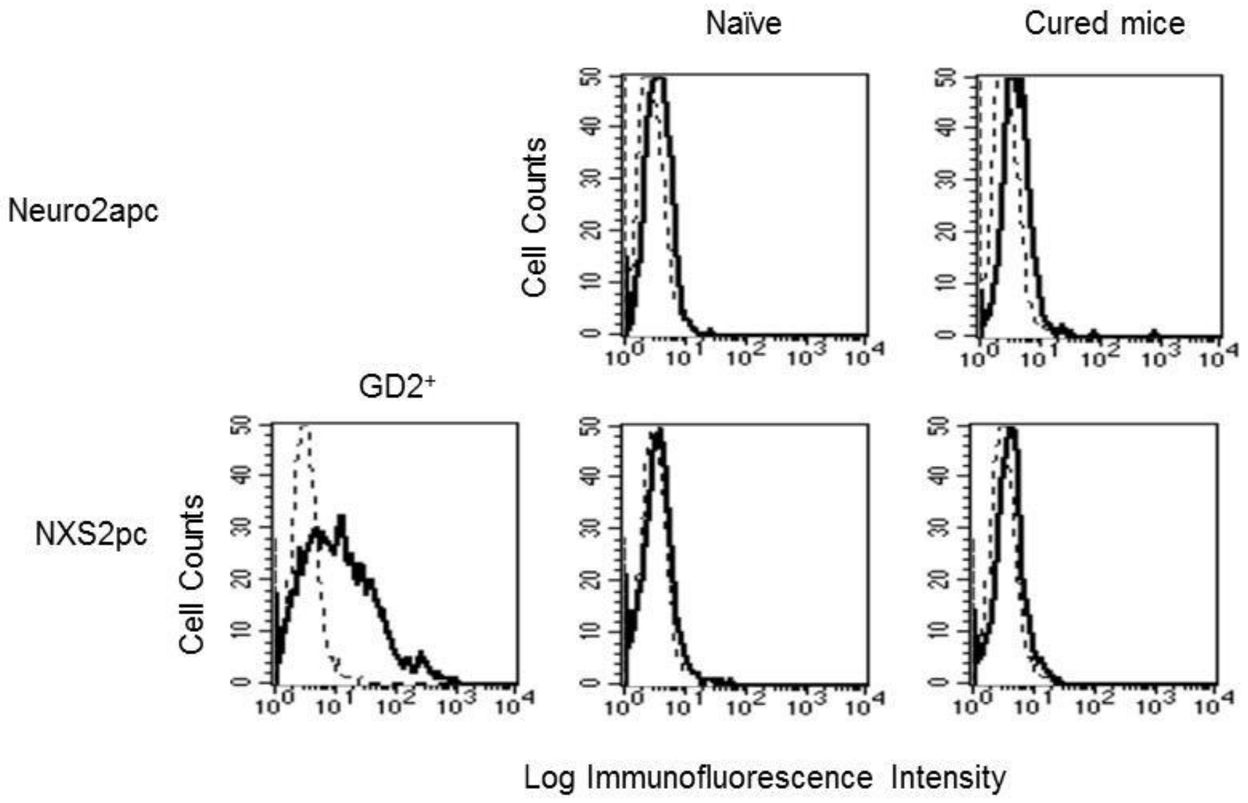

Supplementary Figure S6: Representative FACS analysis of serum samples (1:50 diluted) from naïve and anti-PD-1/anti-CD4 mAb cured mice reacting against Neuro2apc or NXS2pc cells are shown. An anti-total Ig was used as a secondary reagent. Anti-GD2 mAb was used as positive control for the GD2+ NXS2 tumor cells.

Supplementary Figure S7

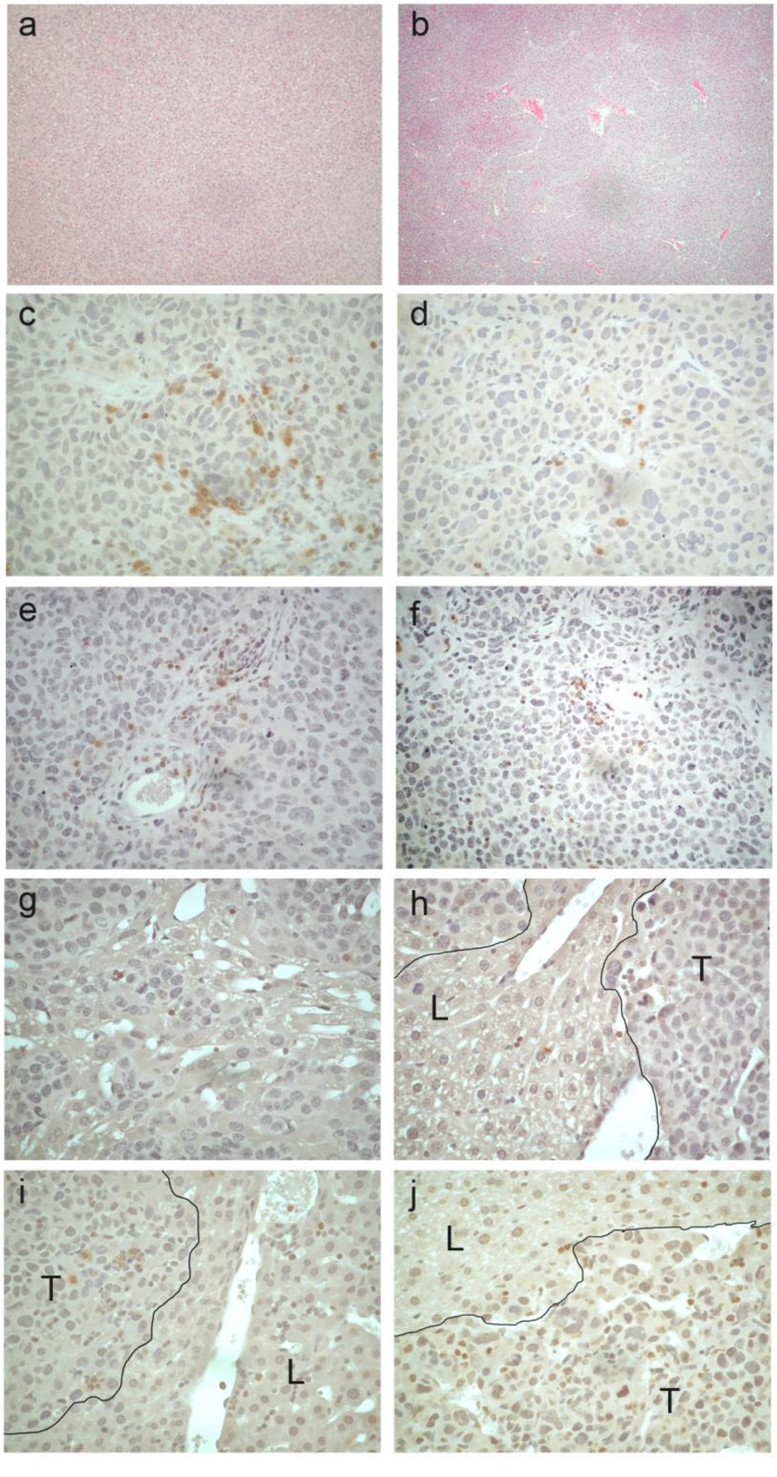

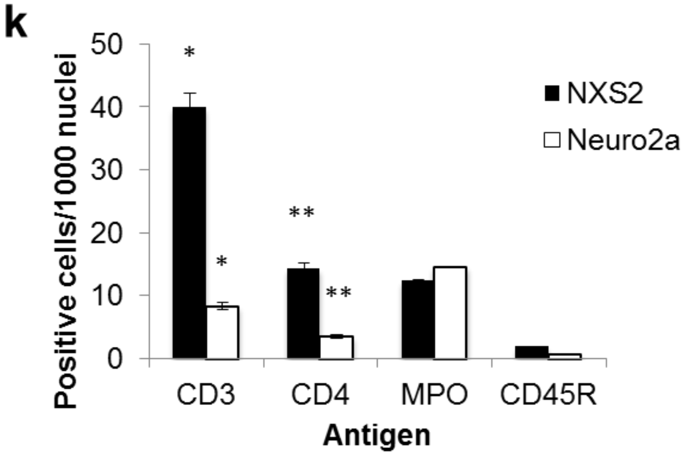

Supplementary Figure S7: Haematoxylin/Eosin staining of liver metastases from NXS2 (**a**) or Neuro2a (**b**) cells. Tumors were morphologically distinguishable, in particular for the greater presence of blood vessels and lacunae in Neuro2a derived tumors. Enlargement 500x. Inflammatory infiltrate in Neuro2a and NXS2 liver metastases. Inflammatory infiltrate was analyzed by immunohistochemistry on paraffin sections. T lymphocytes were evidenced by CD3 staining and, although their distribution within the tissue is not uniform they are more abundant in NXS2 (**c**) tumor respect to Neuro2a (**d**). Same distribution for $CD4^+$ cells (**e**: NXS2; **f**: Neuro2a). B lymphocytes, recognized by B220 staining were rare and mostly outside or at the periphery of the tumors (**g**: NXS2; **h**: Nauro2a). Myeloperoxidase (MPO) recognizes granulocytes, macrophages and monocytes, and their abundance and distribution was identical for the two cell lines (**i**: NXS2; **j**: Neuro2a). L=liver; T= tumor. Enlargement 1000x. **k:** Quantitative evaluation of intra-tumor inflammatory cells. Cells positive at IHC staining were counted and represented as absolute number/1000 nuclei. T student test: * P=0,03; ** P=0,06

Supplementary Figure S8

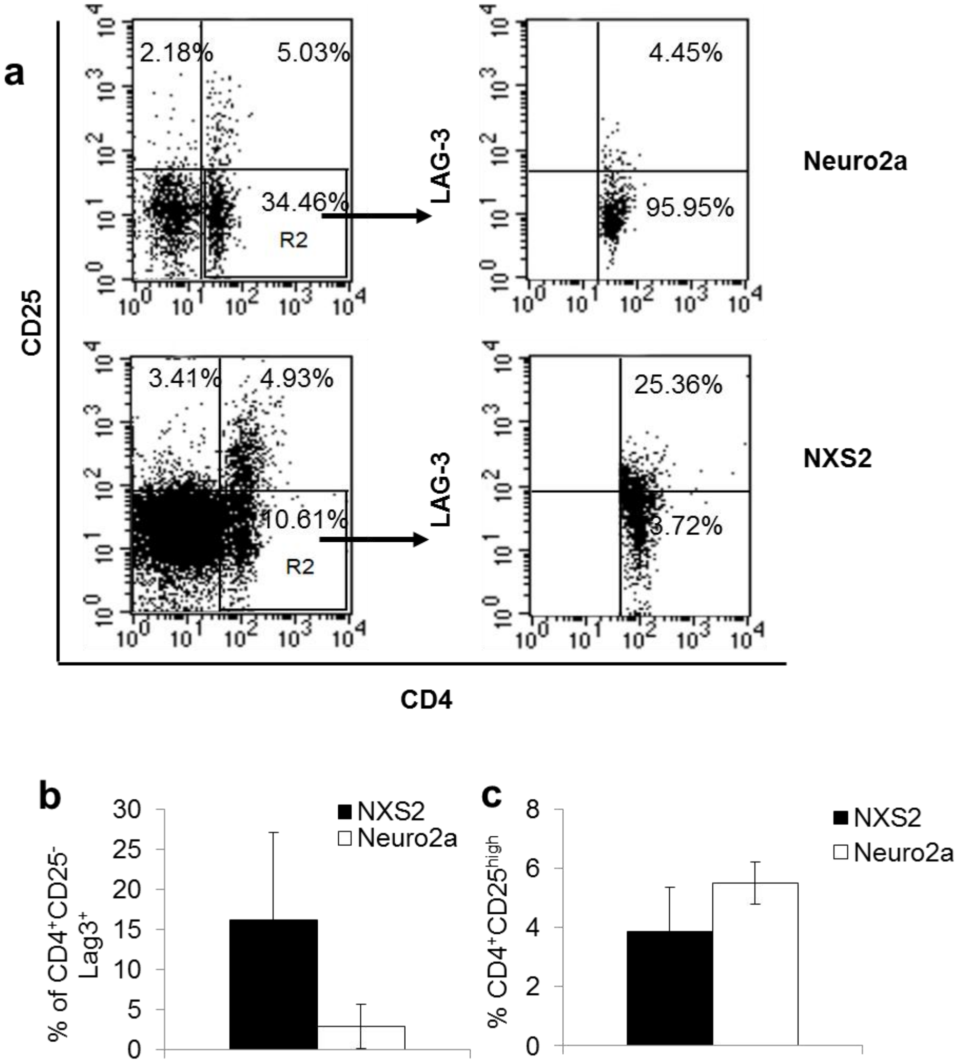

Supplementary Figure S8: a: Representative dotplot of $CD4^+CD25^+$, $CD4^+CD25^-$ T cells (left) and of $CD4^+CD25^+Lag3^+$ T-r1 cells (right) gated on the $CD4^+CD25^-$ population in tumors from untreated or Neuro2a- and NXS2-bearing mice. The percentage of each population is indicated. **b, c:** Graphic representation of $CD4^+CD25^+Lag3^+$ T-r1 (**b**) and Treg (**c**) cells percentages in tumors from Neuro2a- and NXS2-bearing mice. Mean value percentages of double positive cells from 3 mice per group are given.

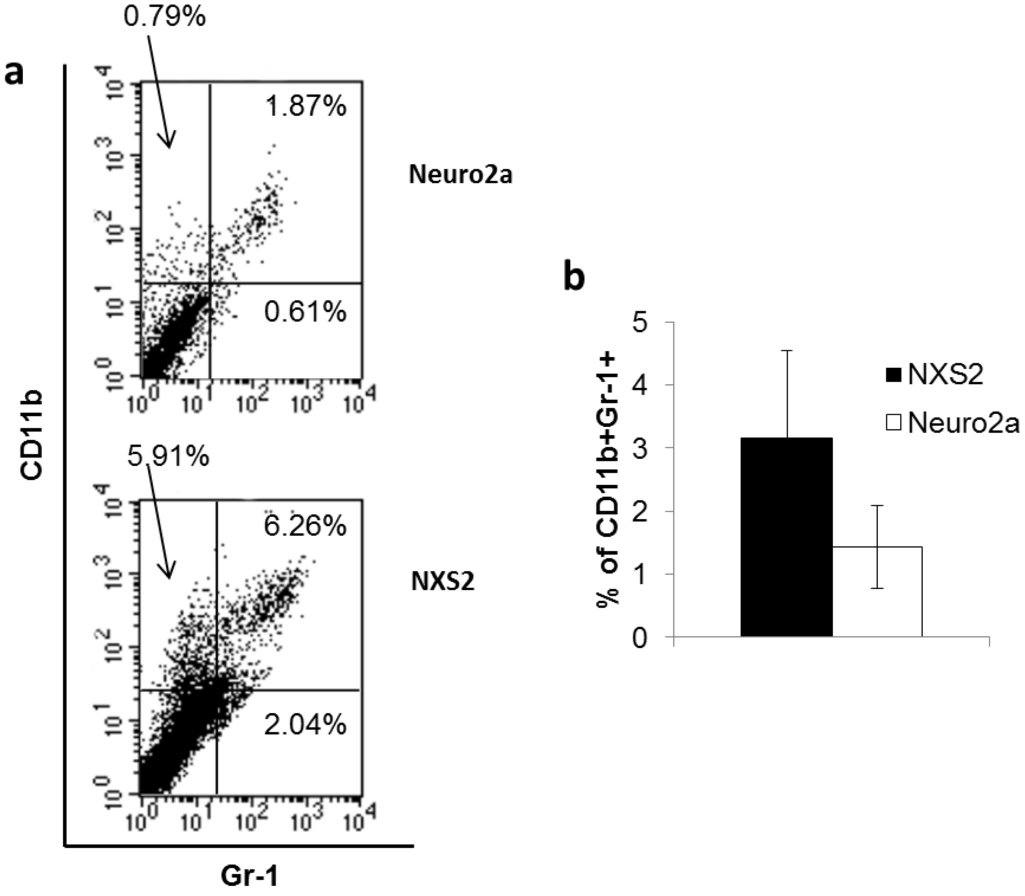

Supplementary Figure S9: a: Representative dotplot of $CD11b^{+}Gr\text{-}1^{+}$ MDSC cells in tumors from Neuro2a- and NXS2-bearing mice treated with anti-CD4 + anti-PD-1 mAb immunotherapy. The percentages are indicated. **b:** Graphic representation of $CD11b^{+}Gr\text{-}1^{+}$ MDSC cells percentages in tumors from Neuro2a- and NXS2-bearing mice treated with anti-CD4 + anti-PD-1 mAb immunotherapy. Mean value percentages of double positive cells from 3 mice per group are given.

Supplementary Figure S10

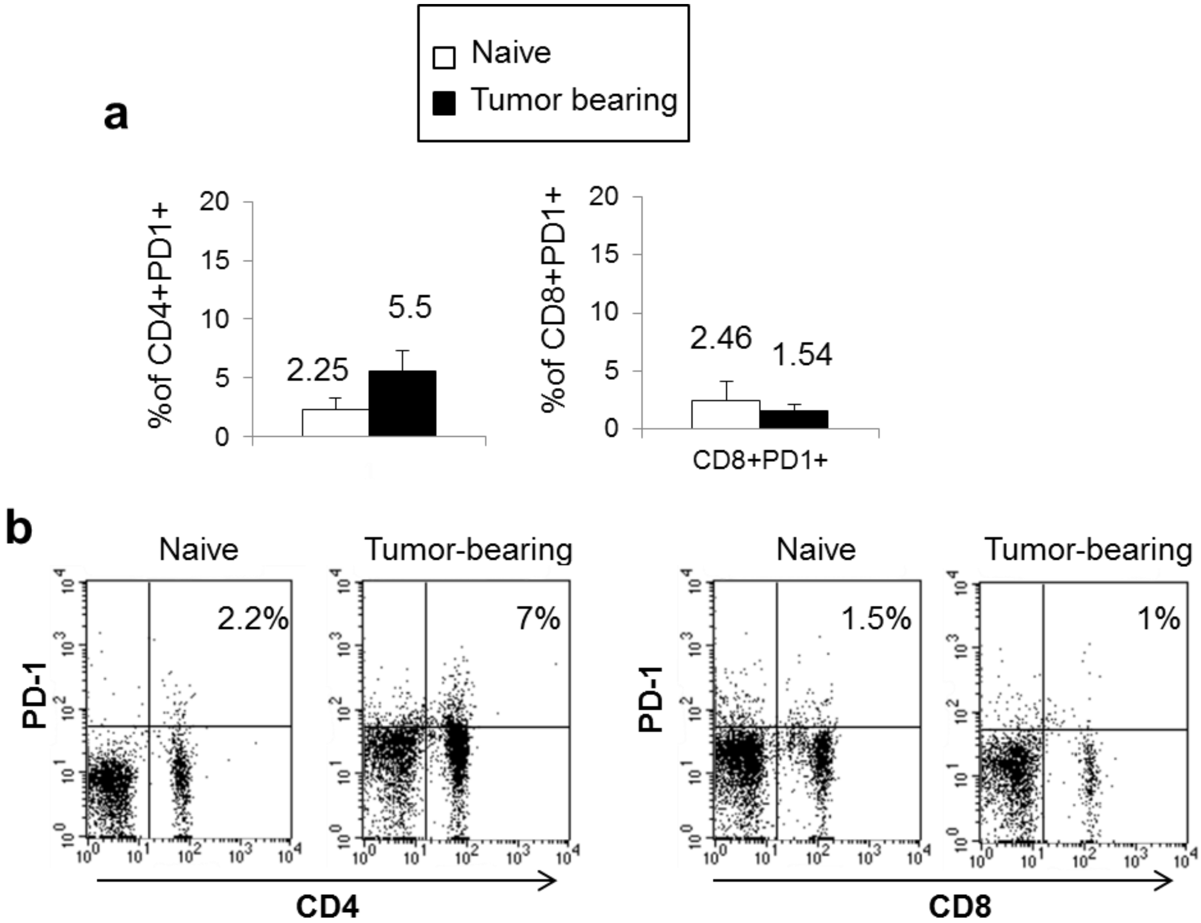

Supplementary Figure S10: a: Percentages of $CD4^{+}PD\text{-}1^{+}$ and $CD8^{+}PD\text{-}1^{+}$ cells in LN from naïve or Neuro2a-bearing mice. Mean value percentages of double positive cells from 3 mice per group are given. **b:** Representative dot plot for naïve and Neuro2a-bearing mice are shown. Percentages of double positive cells are indicated.

Supplementary Figure S11

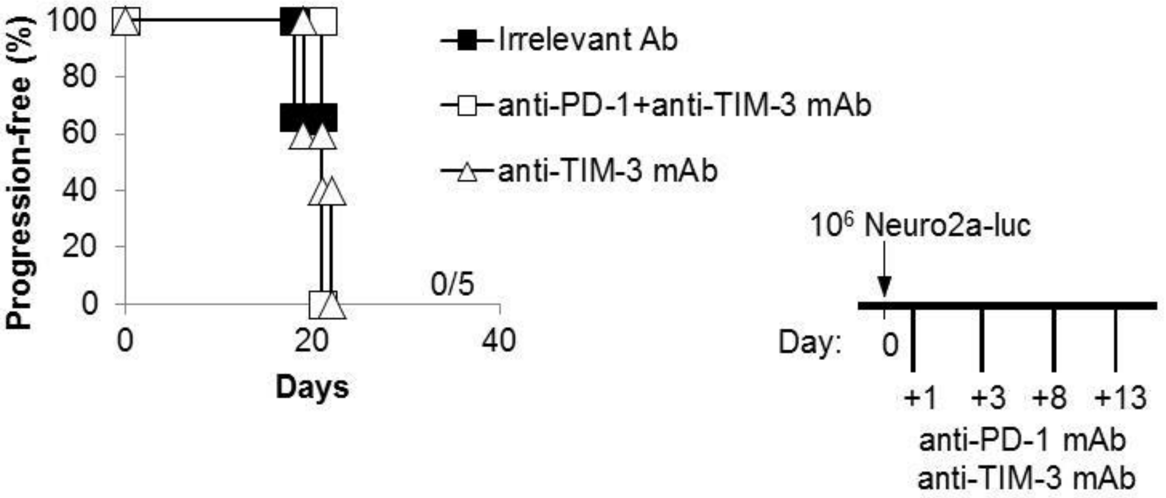

Supplementary Figure S11: Kaplan-Meier analysis of A/J mice inoculated i.v. with a tumorigenic dose of Neuro2a-luc cells on day 0 and treated with an irrelevant antibody, anti-TIM-3 and anti-TIM-3 combined with anti-PD-1 mAb, according to the schedule shown in the inset. Percentages of progression-free mice are indicated on the Y-axis and the fraction of progression-free mice of each group is given in brackets.

Supplementary Table S1

	10 days	19 days	21 days	
	1,28E+07	2,73E+09	4,28E+09	
	5,83E+06	6,35E+09		
Control	1,15E+07	1,61E+09	6,84E+09	
	1,97E+06	4,82E+09	8,65E+09	
	2,20E+06	2,49E+09	9,17E+09	
	10 days	19 days	21 days	40 days
	9,00E+05	4,81E+05	4,22E+05	8,62E+05
Cured by anti-PD-1 + anti-CD4 mAb therapy	1,32E+06	1,24E+06	4,21E+05	6,96E+05
	4,95E+06	2,93E+07	7,69E+05	7,68E+05
	1,61E+06	3,58E+05	4,51E+05	6,86E+05
	7,30E+05	4,08E+05	4,25E+05	9,05E+05

Supplementary Table S1: ROI data of luciferase positive tumors expressed in p/sec/cm2/sr, are indicated for each mouse at the indicated time points.
